# Study of Adaptability and Efficacy of Menstrual Cups in Managing Menstrual Health and Hygiene: A Descriptive Longitudinal Study

**DOI:** 10.7759/cureus.29690

**Published:** 2022-09-28

**Authors:** Ritu Singh, Mukta Agarwal, Sudwita Sinha, Neha Chaudhary, Hemali H Sinha, Monika Anant

**Affiliations:** 1 Obstetrics and Gynecology, All India Institute of Medical Sciences (AIIMS), Patna, IND; 2 Community and Family Medicine, All India Institute of Medical Sciences (AIIMS), Patna, IND

**Keywords:** sanitary protection, longitudinal descriptive study, efficacy, adaptability, menstrual hygiene products, menstrual hygiene, menstrual cups

## Abstract

Introduction

Menstrual cup is a device made up of silicon for menstrual hygiene. Despite its being safe, eco-friendly, cheap, and durable its non-acceptability may be due to higher adoption barriers. We conducted this study to assess the adaptability and efficacy of menstrual cups.

Methods

It was a descriptive longitudinal study, conducted in a tertiary care institute in eastern India. Women of 18-50 years of age, educated till secondary level were included in the study, to be conducted over three months. The quantitative response for the satisfaction with the menstrual cup was measured on a five-point Likert scale after each menstrual cycle. Side effects, the quantity of blood flow and frequency of cleaning the menstrual cup, and how many participants will continue to use it were also asked.

Results

After the third menstrual cycle, 68.9% of participants stated that they would continue the menstrual cup usage. The mean total satisfaction score improved from 5.4 (first cycle) to 12.6 (third cycle) (p<0.001). The majority (67%) had no side effects, 10% had irritation and leakage, and 13% had an unpleasant odor.

Conclusions

The study shows that menstrual cups are a better alternative. Adaptability increases gradually through proper counseling, peer support, and practice.

## Introduction

A woman's life is not so easy managing her home, work, and family at a time. Adding hormones to that is quite a task. Women consider the topic of menstrual hygiene as taboo and hesitate to discuss their feelings or experiences. Yet, among women aged 13 to 51 years who menstruate, the average period lasts three to seven days per month, 6.25 years (2,280 days) over a lifetime. During that time, over 10,000 tampons and pads are used once and thrown away, which is not an eco-friendly thing [1].

The menstrual cup is a reusable, non-toxic, and non-allergic silicone device that can be used to capture menstrual fluids made up of silicon that is non-allergic and not toxic. After insertion of the menstrual cup, it opens in an oval shape and has to be positioned between the posterior fornix and pubic bone, covering the cervix. To remove it, a finger has to be hooked over the rim behind the pubic bone [2].

Menstrual cups have been available for decades, but their use remains limited. Despite its safety, eco-friendliness, affordability, and durability, several barriers to adoption persist. Thus, this study was conducted with the primary objective to assess the adaptability of menstrual cups by examining the level of satisfaction among sexually active women. The study also intends to examine the efficacy of menstrual cups in terms of side effects experienced and perceived ease of usage by the women in the study.

## Materials and methods

The current study adopted a descriptive longitudinal study design to address the research question “do the women find the utilization of menstrual cups satisfactory and effective in reference to its side effects and ease of usage over the period of three months?'' The study was conducted in a tertiary care teaching institute in eastern India after obtaining approval from the Institutional Research Committee of AIIMS, Patna with approval no. IRC/2020/512.

Participants

We enrolled participants in the study conducted over three months. Any sexually active women between 18 and 50 years of age, educated till secondary level were included in the study. Women having allergy or sensitivity to silicone, having any vaginal or urogenital infection, and who had not given consent were excluded from the study.

Sample size

Taking absolute precision (L) of 10%, 95% level of confidence, prevalence (P) regarding usage of menstrual cup 82% [3], and Q as 18% (1-P). The sample size came out to be 59 by applying the formula for single proportion, n=4 PQ/L^2^. However, considering 10% loss to follow-up during the study, a somewhat large sample size of 66 was included in the study.

Study procedures

**Figure 1:**
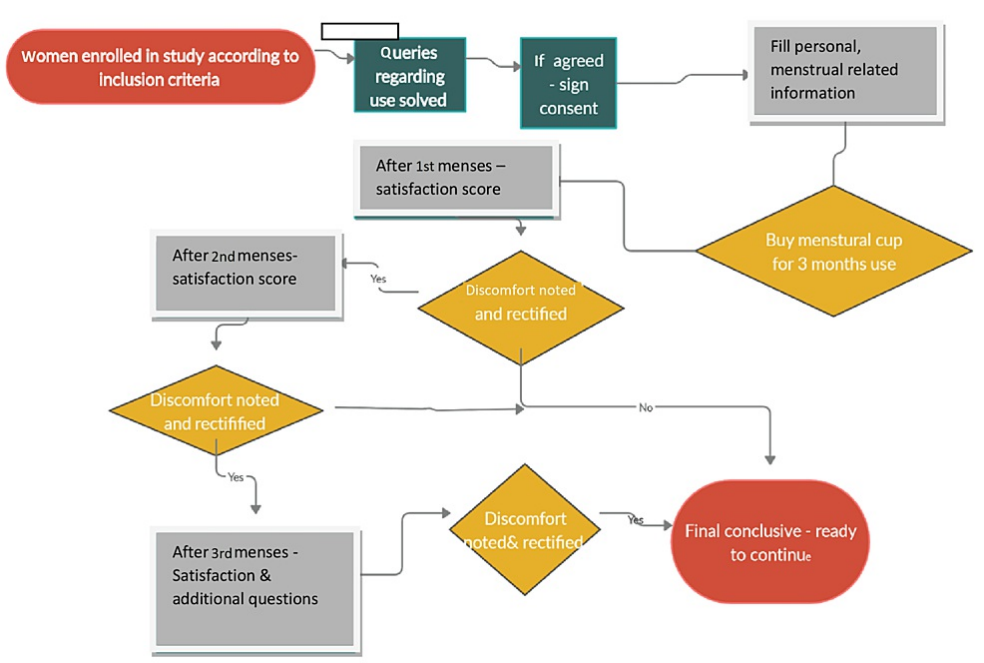
Methodology of adaptability and efficacy of menstrual cup.

Women fulfilling inclusion criteria had been given detailed explanations/information about menstrual cups and the study in the form of a PowerPoint presentation, in a group of 10 women. After agreeing to participate in the study, written consent was taken from them. Further queries regarding the use of menstrual cups had been discussed and solved by video sharing and personal interviews with the women. To collect personal and menstruation-related information a pre-designed questionnaire was administered through personal interviews with respondents. The participants used the menstrual cup for three consecutive menstrual cycles and reported to investigators after completion of each menstrual cycle (Figure 1).

At every visit, participants were interviewed using a structured questionnaire for assessing their satisfaction. Satisfaction with the usage of the menstrual cup was elicited by asking questions regarding the ease of wearing, removing, cleansing, and comfortable feel with the menstrual cup. The quantitative response for satisfaction with the usage of the menstrual cup was measured on a five-point Likert scale, i.e., not at all, slightly, moderately, very, and extremely [4]. The maximum and minimum score for the satisfaction scale was 16 and 0, respectively.

If participants were not able to report every month, they were contacted through telephonic interviews. Also, any difficulty or discomfort experienced by the participants was noted and rectified. During the last visit of participants, i.e., after the third menstrual cycle, additional questions were asked regarding whether they would continue using it or experiencing any side effects (e.g., leakage, odor, and irritation) of the menstrual cup during the study period. Also, they inquired about the quantity of blood flow and frequency of cleaning the menstrual cup.

Statistical analysis plan

The collected data were entered, cleaned, and coded in MS Excel. For presenting the qualitative response of the satisfaction scale variable after each cycle, the responses in terms of slightly and moderately were merged as moderately whereas, very and extremely were merged as extremely. Continuous data were presented as mean and standard deviation. Whereas, categorical data were presented as frequency. One-way ANOVA was applied to detect significant differences between the continuous variables. All the statistical analysis was done using STATA version 13 software (College Station, TX: StataCorp LP).

## Results

A total of 66 participants were recruited for the study to be followed for three consecutive menstrual cycles. Although two participants dropped out after the recruitment, one moved out of the city, and the other one lost to follow-up. Thus, 64 participants reported after the first menstrual cycle. Furthermore, after the first menstrual cycle three participants dropped out, two due to messy feelings, and another one was not able to insert a menstrual cup after repeated attempts. So a total of 61 females were reported after the second and third menstrual cycle.

Table 1 presents the sociodemographic characteristics of the participants noted at the time of recruitment. The majority of females (80.3%) were more than 30 years of age. Maximum participants were graduates (77.3%) and more than half (60.6%) were in the upper-middle class. Approximately, half of the participants (51.5%) were spending $6.81-20.43 annually on buying sanitary protection followed by more than $20.43 (28.8%).

**Table 1:** Sociodemographic characteristics of participants (n=66). *Modified Kuppuswamy scale.

Characteristics		Frequency (%)
Age (years)	<30	53 (80.3)
	>30	13 (19.7)
Education status	Up to class 12	5 (7.57)
	Graduate	51 (77.3)
	Postgraduate	10 (15.15)
Socioeconomic status*	Upper class	15 (22.7)
	Upper middle	40 (60.6)
	Middle	11 (16.7)
Income spends on buying the sanitary protection product in a year	Below $6.81	13 (19.7)
	$6.81-20.43	34 (51.5)
	More than $20.43	19 (28.8)

Table 2 shows the baseline information regarding the practices and perceptions related to sanitary protection. The majority of females used a sanitary pad (95.5%) and more than half (60.6%) were fed up with changing the sanitary pad/tampon/cloth. Approximately two-thirds of females (63.6%) answered they are fed up with disposing of pads or washing used clothes and also majority (90.9%) mentioned that they want some other alternative.

**Table 2:** Baseline information regarding practices and perception related to sanitary protection (n=66).

Characteristics		Frequency (%)
Sanitary protection used	Cloth	1 (1.5)
	Tampon	2 (3)
	Sanitary pad	63 (95.5)
Are you fed up with changing sanitary pad/tampon/cloth?	No	26 (39.4)
	Yes	40 (60.6)
Fed up with disposing of sanitary pad/tampon/cloth?	No	24 (36.4)
	Yes	42 (63.6)
Want some other alternative?	No	6 (9.1)
	Yes	60 (90.9)

First feedback received after the first menstrual cycle revealed that wearing (7.8%), removal (12.5%), and cleaning (17.2%) of menstrual cups was extremely easy. Also, 7.8% found the usage of menstrual cups extremely comfortable. Second feedback as compared to the first feedback noted an increase in the proportion of females who found menstrual cup wearing (29.5%), removal (32.8%), and cleaning (29.6%) extremely easy as well as extremely comfortable (29.5 %) to use. Furthermore, on third feedback, the majority of females reported that menstrual cup was extremely easy to wear (75.4%), remove (77%), clean (78.7%), and extremely comfortable (70.5%) (Table 3).

**Table 3:** Feedback of participants regarding usage of menstrual cups after each cycle. *Number of participants (n) = 64. **Number of participants (n) = 61.

Characteristics	First cycle, n (%)*			Second cycle, n (%)**			Third cycle, n (%)**		
	Not at all	Moderate	Extremely	Not at all	Moderate	Extremely	Not at all	Moderate	Extremely
Easy wearing	23 (35.9)	36 (56.3)	5 (7.8)	10 (16.5)	33 (54)	18 (29.5)	1 (1.6)	14 (23)	46 (75.4)
Easy removal	7 (10.9)	49 (76.6)	8 (12.5)	7 (11.5)	34 (55.7)	20 (32.8)	1 (1.6)	13 (21.3)	47 (77)
Easy cleaning	11 (17.2)	42 (65.6)	11 (17.2)	5 (8.2)	38 (62.2)	18 (29.6)	2 (3.3)	11 (18)	48 (78.7)
Comfortable	11 (17.2)	48 (75)	5 (7.8)	7 (11.5)	36 (59)	18 (29.5)	4 (6.6)	14 (23)	43 (70.5)

Table 4 shows the gradual acceptability of menstrual cups noted among the participants which was evident from the statistically significant improvement in the mean score of the satisfaction scale after every menstrual cycle. The mean total satisfaction score improved from 5.4 (first cycle) to 7.7 (second cycle) and 12.6 (third cycle) (p<0.001).

**Table 4:** Mean score of the satisfaction scale after each of the three consecutive menstrual cycles.

Characteristics	First cycle, mean (SD)	Second cycle, mean (SD)	Third cycle, mean (SD)	F, p-value (ANOVA)
Easy wearing	1.1 (0.09)	1.9 (1.2)	3.2 (1.1)	58.8, <0.001
Easy removal	1.4 (0.9)	2 (1.03)	3 (0.9)	39.9, <0.001
Easy cleaning	1.5 (1.1)	2 (1)	3.3 (1.1)	44.6, <0.001
Comfortable feel	1.4 (0.9)	1.8 (1)	3.1 (1.3)	39.2, <0.001
Total satisfaction score	5.4 (3.5)	7.7 (3.9)	12.6 (4.2)	56.7, <0.001

**Figure 2:**
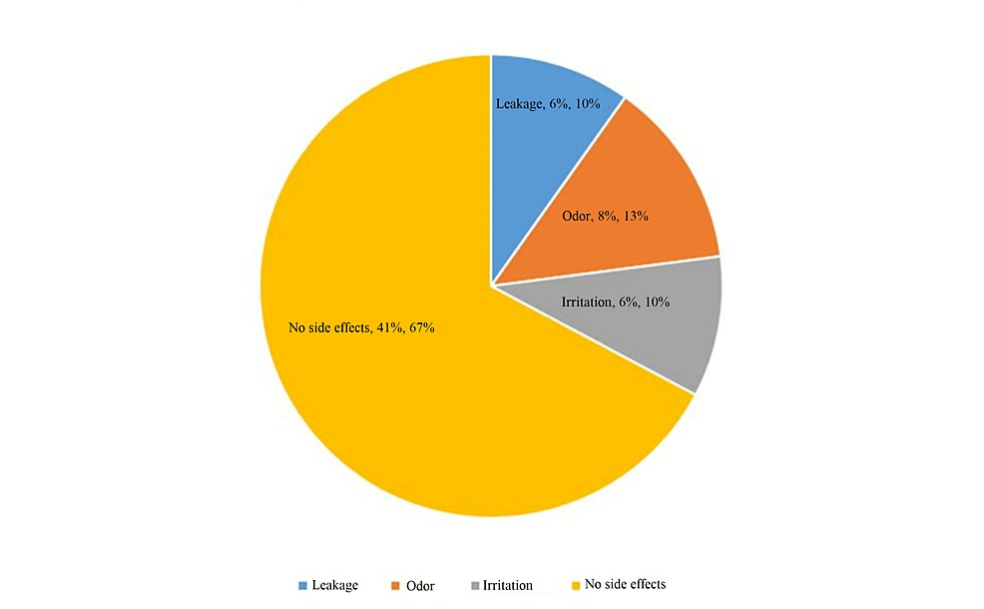
Side effects of menstrual cup after the third menstrual cycle.

**Figure 3:**
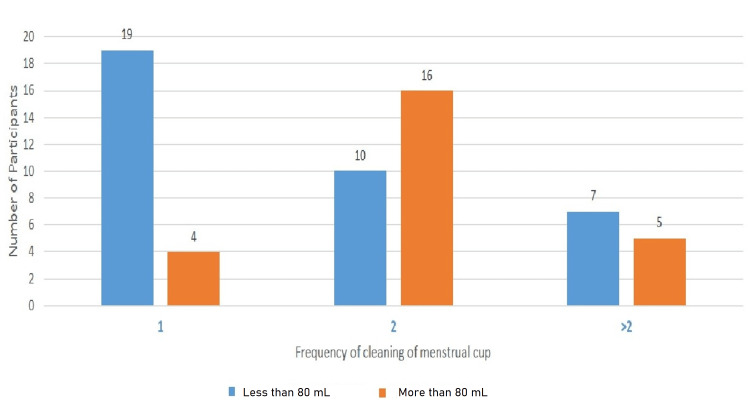
Quantity of blood loss and frequency of cleaning the menstrual cup (n=61).

Figure 2 presents the side effects of menstrual cups noted after the third menstrual cycle. The majority (67%) of females mentioned that after usage of menstrual cups they experienced no side effects, 10% of participants had irritation and leakage, and 13% of participants had an unpleasant odor. Maximum participants 36 (59%) had cleaned menstrual cups two times a day, in which 10 had blood loss of less than 80 mL and 16 had more than 80 mL (Figure 3). After the third menstrual cycle, 68.9% of participants stated that they would continue menstrual cup usage for sanitary protection.

## Discussion

The menstrual cup is a device made up of silicone that is placed inside the vagina under the uterine cervix to collect the menstrual blood [2]. In 1867 in the United States, the first models, named "catamenial sacks" were patented [5]. And then years later, in 1937, the first commercial prototype was patented by Chalmers [6]. Initial acceptance of cups was not good, but in the wake of the "tampon crisis" due to the toxic shock syndrome cases, the cup had its comeback in the 1980s [7]. At first menstrual cups were made of latex, but due to frequent allergic reactions led to their removal [8]. Finally, in 1998, with the arrival of hypoallergenic medical silicone, it has become the material of choice until now [9].

However, despite their long history in the market, many women are still unaware of their existence [10]. It may be because of the higher adoption barrier of menstrual cups. A systematic review and meta-analysis published in Lancet in 2019 [11] states that in all qualitative studies with practice, peer support, and training there will be familiarization with the menstrual cup over time [12-14]. A study done in Kerala, India, in 2022 also showed that in participants insertion and removal became significantly easier after third-time use onwards [15].

In low-income and middle-income countries, longitudinal quantitative studies showed a learning curve of two-five months [11]. As consistent with our study, constant training and practice made women comfortable with using the menstrual cup.

In 15 studies, 73% (pooled estimate: n=1144; 95% CI, 59-84; I^2^=96%) of participants were ready to continue the menstrual cup after the study period of two to five months of use [11]. In a cluster-randomized trial from rural India, the acceptance of menstrual cups was significantly lower than that of pads, 50% by the end of six months [16]. In our study, 68% of participants were willing to continue the menstrual cup after the end of three months. This can be because we have included only those participants in the study who are educated, from urban populations, and willing to participate in the study.

In a UK study compared with tampons or sanitary pads, menstrual cups were having a decrease in the average number of changes per cycle [17]. In our study, 80% of participants changed the menstrual cup one or two times, irrespective of blood loss.

While considering financial and environmental implications, gathering rough calculations over 10 years, purchase costs and waste from a menstrual cup will be a small fraction as compared to pads or tampons, e.g., if compared with using 12 pads per period, use of a menstrual cup would comprise 5% of the purchase costs and 0.4% of the plastic waste, and compared with 12 tampons per period, use of a menstrual cup would comprise 7% of the purchase costs and 6% of the plastic waste [11].

In our study, the use of a menstrual cup would comprise on average, 3.3% of the purchase cost compared with sanitary protection measures in 10 years. The major concern for non-acceptance of a menstrual cup is that it needs manipulation into and out of the vagina and compel contact with genital tissues and fluid. This can be conquered by repeated counseling of the users regarding its use. There is no other sanitary measure than this which makes the quantitative assessment of menstrual blood loss.

In our study, 10% of participants had irritation and leakage and 13% of participants had an unpleasant odor. In a study done by Kakani et al. in Gujarat, among 158 participants rashes, dryness, and infection were noted in a few cup users [18]. In another study, cramping (1%), leakage (1%), and improper fit (3%) resulted in cup discontinuation [19]. The limitation of this study is that it is conducted on a very small scale and large sample size is necessary for generalization of data to general population.

## Conclusions

This study shows that menstrual cups are a better alternative to the current methods of menstrual sanitation as it is durable, eco-friendly, comfortable, safe, have no need for frequent changing in a day, and have no disposal issues. Adaptability increases gradually through proper counseling, peer support, practice, and consistency. Third time onwards insertion and removal become easier for women. After third cycle, 68% of participants agreed to continue using menstrual cups in our study. More randomized controlled trials and long-term prospective cohorts are needed for complication and compliance. Awareness campaigns are also necessary for society.
